# The People Versus the Dentist

**Published:** 1905-05-15

**Authors:** Geo. Zederbaum

**Affiliations:** Charlotte, Mich.


### THE PEOPLE VERSUS THE DENTIST.

BY GEO. ZEDERBAUM, D.D.S., CHARLOTTE, MICH.

Read before the Southwestern Michigan Dental Society, April 11, 1905.

Mr. Chairman, members and friends, of the Southwestern Michigan Dental Society: I am indeed more than honored to receive a request from your secretary to appear before you to-day. Whether this is simply a practical joke of my classmates or possibly your intention to draw me on and then criticise, ridicule and laugh at me, I do not know; however, I selected a subject over which I can laugh with you, and that is: "The Public versus the Dentist."

Although much has been done during the last decade in dentistry to materially improve the relation between the public and the dentist, there is still need for more persevering work in educating the public as to what dentistry really is and how it should be valued; there still remains almost a Herculean task for the dentist who wishes to still further improve the relation that should exist between the intelligent public and his profession. Even at this date there are perhaps no more than two people out of every ten that value or properly care for their teeth; and, as often has been said, each one of them has or should have thirty-two reasons for taking the best care of his teeth, and they are all in his own head.

How often a patient appears in our office, wishing immediate extraction of some offending, or even an offended, member of his masticatory force, when it could be readily repaired for a very small fee. But no amount of persuasion can induce him to have that repairing done, because, as he puts it, "it will have to come out eventually anyway, and I'd rather lose it right now than to pay for filling it and then again pay for extracting it."

Another one comes in and says to you, "yank it out quick, doctor; it is decaying from the inside,'' and when the usual lengthy story that ever since he had his teeth cleaned by a dentist they commenced to rot right down and he cannot masticate his food any more.

Still another may say that he'd rather lose his tooth than to sit in that dental chair two minutes and hear that buzz-saw.

These and many similar, and, to us, nonsensical reasons are heard almost daily in offices of a mixed clientele.

Only recently I had an appointment with a fifteen-year-old girl, whose mouth was simply filthy; many teeth requiring attention. She positively refused to keep the appointment, and her mother brought her up in spite of her wishes. Of course the first thing I wanted to do was to clean her teeth as thoroughly as possible, but even that was out of the question, as she would not let an instrument touch her teeth, and the sight of the engine completely unnerved her. No amount of persuasion or explanation would do. As luck would have it, my wife brought my two-and-a-half year old daughter to the office just at that critical moment, and I immediately asked the young miss to vacate the chair just for a few moments and called to my little one; she at once climbed into the chair, I fastened the napkin and went to work as if her little teeth were in actual need of the procedure. She has been there before and did not mind the scalers nor the engine a particle. After the work was finished my fifteen-year-old patient consented to go through the same course, and, mind you, without even a whimper from her. I gradually progressed toward the harder work and am now devitalizing a tooth for her. The old saw, "Example is better than precept," proved true in this case, as it has in many others.

On the other hand, we encounter many cases which are exactly the opposite. Some patients can not possibly think of losing a tooth, even a nasty, abscessed root; they had rather go on paying doctors and druggists' bills to the amount of several times the necessary cost of relieving their lamentable condition, by judicious extraction of irreparable teeth and placing the mouth in a healthy condition. I know of two cases in my own practice where both patients had been advised to have immediate extraction of every tooth, if I may designate such remnants as teeth; instead, both cases have been under the constant care of physicians for treatment of chronic stomach affections, submitting to daily use of the stomach pump, and dieting on hot water and toast. At last they both were obliged to return to me for relief, which was swiftly brought about by the extraction advocated a year previous.

Then again we very often advocate an extraction of some irreparable tooth and the patient exclaims, "No, No! I'll wait 'till it aches, than I shall have more grit to have it out," and in most cases that is just the time when they have the least, and the tooth crushes like an egg-shell even in the hands of the best extractors.

Occasionally a patient comes in with his mind fully made up to have a tooth removed, and same might be necessary, but he is trembling in his boots because he once heard some one had a jaw broken in the extraction of a tooth. Patient, knowing nothing further, is strongly impressed by such talk, and regards the operation as a very serious affair, not knowing that a spicula of the alveolus may come away in extraction of any tooth. In this connection I can cite an amusing incident that occurred in my practice last summer. A young physician practicing in a small country four-corners, called with an aching lower third molar, which, under existing condition, I found advisable to extract. The man had his mind fully made up to that very expedient and the deed was done with no difficulty whatever, the tooth coming away clean and clear. The doctor, who was one of those big, fat and flabby sort of chaps, with no more nerve as far as his own personal injuries are concerned than a jelly fish, carried on quite considerable from what he termed his "after-pains." I placed a loosely-rolled pledget of cotton saturated with chloroform into the vacated socket, with instructions to remove it within a half hour. I expected him to be equal to that, but it seems he was not, as he at once went to a brother physician who promptly removed it and packed the alveolus with morphine. This, as might be expected, coming in contact with raw tissues raised havoc, general irritation and then much swelling, sloughing and pain set in, and, what perhaps worried him more than all else, it became impossible for him to open his mouth. These symptoms were exceedingly aggravating because the fact that he was a groom of only two week's standing and naturally wanted to be petted and to learn how much his young wife really loved him. She at once played the ministering angel act and with tears in her eyes sent him to bed. It was then that I received a hysterical telephone message that I am sure must have melted the wires at either one end or the other. I left my office with my assistant, hired a conveyance and drove out to his home expecting to find a corpse; but, thank heaven, he was not dead yet! I examined his mouth as best I could and nothing serious appeared to be the matter, he was not in any worse condition I may safely say than every one of us present could expect under the circumstances. On their way home their horse ran away, but they finally, after much delay, got home in the midst of a terrific rain storm, wet through to the skin and considerably shaken up. I left some echafolta solution to be used locally, advised hot, moist dressings and departed. Imagine my surprise, when the next issue of the local paper came out, to see that my doctor patient was in bed suffering from a broken jaw resulting from the extraction of a wisdom tooth by a local dentist. I contradicted this report in the next issue, using no name, as mine had not been mentioned, and hushed the matter in this way as best I could; but it was several months before I had a patient come into my office to have a tooth extracted who did not tell me about that case of which he had read, and wondering what dentist it was. I never denied the fact that it was I who extracted the tooth for the doctor, and I took particular pains in each case to explain the circumstances as just described. Shortly after this incident an old man, a neighbor of the doctor's, came in to have some seventeen teeth extracted, and, to use his dialect exactly, I'll quote: "Say, you're the dentist that broke Doc's jaw, hain't you?" here I started to explain that the jaw was not broken, but he interrupted by continuing, "well I am golldarn glad of it, put it there," he grabbed my hand, squeezed it and jumped into my chair. "Say, Doc., you go right ahead and take out all of mine, will you?" I afterward learned that the doctor had broken off an upper molar for him in the attempt to extract it and caused him excruciating pain for which he'll never forgive him. "It is an ill wind that does not blow some one some good." I'll give the doctor the benefit of saying that he had no intention whatever of injuring my practice, as he has sent a good many my way. His item was simply to show how brave a hero he was to meet so many serious accidents in one day and still live.

But this little episode clearly proves how easily it is to alarm the whole community by just a few injudiciously-used words, and perhaps damage a practitioner's reputation.

Occasionally a patient will come in for plates, and upon examination you find may be two, usually only one tooth on either jaw, around which they wish you to build the plates. You try to demonstrate why such a plate would be impracticable, and why the teeth should be taken out, but it is very seldom that you will succeed in having them part with their old comrades. They know a plate of that sort is harder to construct, and seem to think that is just the reason you argue against it.

Sometimes a patient presents himself for examination, having been elsewhere for the same purpose not over ten minutes before. He distinctly remembers what the other dentist advocated and what his estimate for the work was. He sits down in your chair and acts as if he never saw one before. You examine carefully and your name is "mud" if you find one more cavity than the other dentist, or if your estimate is a dollar higher.

Occasionally a patient comes in knowing exactly what he wants to have done; his teeth may not need or be adaptable for the work he has planned, and here again you are in trouble, for no amount of persuasion will change his ideas and he will go to try his luck elsewhere, and have it all done as he planned, where the dentist says "yes" to everything.

It is not in all cases that patients disagree on financial questions, oh, no! This patient may want two or three anterior superior teeth gold-crowned (thinks he'll look richer), when his teeth could be easily porcelain or gold-filled and appearance made decidedly more neat and esthetic. Should you refuse to do it, he will keep on trying for he is sure to find some dental parlor where they will be tickled to do it while he waits.

Sometimes a grouchy father will bring up his ten-year-old daughter whose teeth are in a sadly neglected condition. After he hears your opinion as to work and learns the probable cost, he'll be apt to say like one of my patients told me, "Well, no, I am not going to spend that on her teeth; they will have to be yanked out before long anyhow, and I'll tell you right here (pointing to his upper plate), I would give more for this plate than for all the teeth that I ever had filled." He evidently intends to have his daughter lose her teeth as quickly as possible so that she can have a plate at fifteen. In this particular case, however, the man is being fooled, for his wife is saving every penny, bent on having her daughter's teeth fixed properly; and, as we all know, when a woman determines to do a thing she'll do it at all hazards. Her husband was simply ignorant and stingy, as many of our patrons are. I recall a case where a middle-aged man came into my office one day and handed me several fragments of an upper plate, telling me that he had the misfortune of breaking his plate. It could not be repaired, so I advised a new plate. He insisted that I use the old teeth. Upon examining his mouth I easily noticed the man had never worn a plate and, after pumping him for a while I learned that the plate belonged to his mother-in-law, who had died recently and he thought he could make use of her at last by rebuilding her teeth for himself. Could any one be meaner than that?

Only the other day it came to my knowledge that a young lady for whom I was working had left her home to work so as to earn enough money to repair her naturally beautiful teeth, as her father positively refused to give her any money to squander on her teeth, as he had never seen a filled tooth or one without a nerve that was worth anything.

Summing it all up, how are we to improve such conditions? What shall we do to better educate and enlighten the average community? Are the people always to blame? Oh, no, not by any means, dentists themselves are largely at fault. What would you do if you were informed by one tailor that the checkered stuff is up to date just now, and another, just across the way, tells you that plain goods are all the rage? You surely would say one or the other or perhaps both are lying. This may truly apply to us. I may advocate a Richmond crown for some tooth; while the dentist next door says by all means put on a gold one; one across the street says extract the tooth; and still another says he can devitalize and fill it. What is the poor patient to do? Here he has had four different opinions from four perhaps the best dentists in town inside of perhaps that many minutes. Gentlemen, how are we to improve such deplorable conditions? Most of us are depending upon our practice to maintain our existence, and we give advice according to our ability, and it may not always be to the best interest of our patient, which of course is decidedly wrong. If I am not good at making a bridge, but am fairly successful at partial plate-work, in all probability I should advise and even strongly argue for the partial plate, when I know full well that the bridge would be better. Where is the secret of such vast differences of opinions?

It is true we come from all over the universe, but it is equally as true that the teachings of our dental colleges never vary to such an extent as to admit of vast discrepancies. The opinion as to what is the best for a certain case should not vary much, and our fees, especially in same locality, should be as nearly as possible the same.

When the patient realizes that several good dentists give similar advice and the charges are nearly the same, we have accomplished a good deal toward his education.

In some towns I could mention, the dentists hold occasional meetings, establish fees from which they depart even within a few days. Such actions are unpardonable, and serve not only to injure the profession as a whole, but certainly prove in time ruinous to the under-bidder. My motto is to charge fair fees for all that I do, and do the work with painstaking accuracy. If there should come a piece of work that you can not do properly, rather than advise something in substitution for the best, send your work to a good laboratory, and you will not cause any unjust injury to your patient. It is my belief that patients ought to receive a short but concise explanation as to what their work ought to be, and how and why certain steps are necessary to so complete it. It is almost daily that I show a little chart of Dr. Bannister's, which no doubt, most of you have seen. This little diagram shows the roots of the teeth and their respective nerves; it takes but a minute (and none of us are so busy that we can not stop a minute to explain), and the patients see for themselves and understand more clearly what is to be done and they are more satisfied. I say, we owe it to them; the teeth are theirs and they should be consulted and educated. No surgeon ever performed an operation without first explaining to either the patient or his next of kin why it should be done and in a few words, using no big technical terms, explain how same is to be accomplished. How much easier this explanation can be made by us dentists when we have charts, drawings, photographs and plaster models of teeth with cavities prepared in them or even by samples of finished work. Patients, as a rule, absorb all this and feel more friendly and are decidedly more composed. It is not true that the dental knowledge on the part of laity hurts the dentist in anyway. The man who tries to make you believe it is for your interest that your patrons be kept in ignorance of all that pertains to their respective cases, must have some other reason than the interest of the profession or the patient for what he says. If you scan through the list of your patrons you will realize at once that the more intelligent they are the less trouble you have with them, and the more they appreciate your services. And so we must work with honesty of purpose; we must have energy, ambition and life in our work. "Dead men tell no tales," you all know the old proverb; brought down to date it might be made to read, "dead men make no teeth." But there is such a thing in life as dying by inches without showing the common symptoms of decay; men sometimes totter to the grave unknowing themselves that their end is near. Nothing can be sadder than a case of this kind. Keep alive, put your whole soul into your work and the day will be shorter whatever the task. No day is too long for honest effort, honestly put forth. We must educate our old patients as well as our new patients; our young and our aged patients. The day may come when our efforts will be realized by some future generation of dentists; and, if it were possible for us to return, then, no doubt, we would be more than glad to continue the work of our lives, as we all love our profession and we all must love our fellow-men.

"Abou Ben-Adhem—may his tribe increase—
Awoke one night from a sweet dream of peace,
And saw, within the moonlight in his room,
Making it rich and like a lily in bloom,
An Angel writing in a book of gold.
Exceeding peace had made Ben-Adhem bold,
And, to the presence in the room, he said:
'What writest thou?' The vision raised its head,
And, with a look made all of sweet accord,
Answered: 'The names of those who love the Lord.'
'And is mine one?' asked Abou. 'Nay not so,'

Replied the Angel. Abou spoke more low
But cheerily still, and said: 'I pray thee then,
Write me as one who loves his fellow-men.'
The Angel wrote and vanished. The next night
It came again with a great awakening light
And showed the names whom love of God had blessed,
And lo! Ben-Adhem's name led all the rest.''

